# Computing phenomenologic Adair-Klotz constants from microscopic MWC parameters

**DOI:** 10.1186/1752-0509-3-68

**Published:** 2009-07-14

**Authors:** Melanie I Stefan, Stuart J Edelstein, Nicolas Le Novère

**Affiliations:** 1 Computational Neurobiology Group, EMBL-EBI, Wellcome-Trust Genome Campus, Hinxton, CB10 1SD, UK

## Abstract

**Background:**

Modellers using the MWC allosteric framework have often found it difficult to validate their models. Indeed many experiments are not conducted with the notion of alternative conformations in mind and therefore do not (or cannot) measure relevant microscopic constant and parameters. Instead, experimentalists widely use the Adair-Klotz approach in order to describe their experimental data.

**Results:**

We propose a way of computing apparent Adair-Klotz constants from microscopic association constants and allosteric parameters of a generalised concerted model with two different states (*R *and *T*), with an arbitrary number of non-equivalent ligand binding sites. We apply this framework to compute Adair-Klotz constants from existing models of calmodulin and hemoglobin, two extreme cases of the general framework.

**Conclusion:**

The validation of computational models requires methods to relate model parameters to experimentally observable quantities. We provide such a method for comparing generalised MWC allosteric models to experimentally determined Adair-Klotz constants.

## Background

Quantitative descriptions of biological processes are one of the main activities in Life Science research, whether in physiology, biochemistry or molecular and cellular biology. They offer a way of characterising biological systems, measuring subtle effects of perturbations, discriminating between alternative hypotheses, making and testing predictions, and following changes over time. There can be many different ways to describe the same biological process. Phenomenological descriptions provide a way of relating input and outcome of a given process, without requiring a detailed knowledge about the nature of the process or possible intermediate steps. Since they provide a direct link between input and output, they can be easily applied to experimental results. On the other hand, Systems Biology favours more mechanistic representations, that aim at exploring how exactly behaviours of systems emerge from intrinsic properties and interactions of elements at a lower level. Using the former descriptions to build and validate the latter representations may prove a challenge in some cases.

Several types of descriptions may co-exist for a given biological problem. One of these problems is the binding of ligand to a protein with several binding sites, and the apparent cooperativity observed in this context, for which various frameworks have been developed throughout the XX^th ^century [1].

Drawing on observations of oxygen binding to hemoglobin, Hill [2] suggested the following formula for the fractional occupancy  of a protein with several ligand binding sites:

(1)

where *K *denotes an apparent association constant, [*X*] denotes ligand concentration, and *n*_*H *_the "Hill coefficient", intended to be a measure of cooperativity.

Adair [3] and Klotz [4] (reviewed in [5]) further explored the notion of cooperative binding. According to their framework, cooperativity was no longer fixed, but dependent on saturation: There were different binding constants describing binding of the first ligand, the second, the third, etc. It is worth noting that these constants do not relate to individual binding sites. They describe *how many *binding sites are occupied, rather than *which ones*. This framework is often used by experimentalists to describe measurements of ligand binding in terms of sequential apparent binding constants. According to this framework, the fractional occupancy of a protein is given by the following equation [4]:

(2)

Where *n *denotes the number of binding sites and *K*_*i *_the *i*^*th *^apparent association constant

The Monod-Wyman-Changeux (MWC) model for concerted allosteric transitions [6] went a step further by exploring cooperativity based on three-dimensional conformations. It was originally formulated for oligomeric proteins with symmetric, identical subunits, each of which has one ligand binding site. According to this framework, two (or more) interconvertible conformational states of an allosteric protein coexist in a thermal equilibrium. The ratio between the two states (often termed "*T*" for "tense", and "*R*" for "relaxed") is regulated by the binding of ligands that have different affnities for each of the states. For instance, in the absence of a ligand, the *T *state prevails, but as more ligand molecules bind, the *R *state (which has higher affnity for the ligand) becomes the energetically favoured conformation. The constant *L *describes the equilibrium between both states when no ligand molecule is bound: *L *= [*T*_0_]/[*R*_0_]. If *L *is very large, most of the protein exists in the tense state in the absence of ligand. If *L *is small (close to one), the *R *state is nearly as populated as the *T *state. The constant *c *describes the ratio of association constants for the *T *and *R *states for each site: *c *= *K*^*T*^/*K*^*R *^(note that MWC equations are most often expressed with dissociation constants. However, we will use association constant throughout this paper for the sake of consistency with Hill and Adair-Klotz schemes). If *c *= 1, both *R *and *T *states have the same ligand affnity. The *c *value also indicates how much the equilibrium between *T *and *R *states changes upon ligand binding: the smaller *c*, the more the equilibrium shifts towards the *R *state. According to the MWC model, fractional occupancy is described by:

(3)

where [*X*] denotes ligand concentration, and with *K*^*R*^, *L *and *c *as described in the paragraph above. In this paper, we first propose a generalised MWC framework that can be applied to proteins whose ligand binding sites have different affnities. We then develop a set of equations that uses the parameters of such a generalised MWC model to compute apparent association constants according to the Adair-Klotz model. We show how these can be used in order to compare model results with experimental data using two examples which constitute extreme cases of the general framework, calmodulin and hemoglobin.

## Results

### Generalisation of the MWC model

The MWC model can be adapted to describe a protein (whether oligomeric or monomeric) with several ligand binding sites possessing different affinities. In that case, microscopic association constants are termed  and , and their ratio is denoted by *c*_*i *_for the *i*^*th *^binding site.

In this case, the fractional occupancy is described as follows:

(4)

where 1 ≤ *i*, *j *≤ *n*,  and *L *and [*X*] as described above.

If not all binding sites are different, but *m*_*i *_binding sites have the same affinity , identical binding sites can be grouped and the above equation written as:

(5)

where 1 ≤ *i*, *j *≤ *k*, *m*_*i *_denotes the number of binding sites with affnity  (note that Σ_*i*_*m*_*i *_= *n*), and *L*, *c*_*i *_and [*X*] as described above.

Similarly, it is possible to develop generalisations of the equation for fractional conformational change (). In the case of a protein with *n *different ligand binding sites, the corresponding expression is:

(6)

When all  and all *c*_*i *_are equal, this corresponds to the original MWC equation [6].

Again, when binding sites are pooled into groups of *m*_*i *_binding sites that have the same affnity  (where Σ_*i *_*m*_*i *_= *n)*, then  can be written as follows:

(7)

In order to compare the numerical outcomes of their models with experimental results, modellers using either the original or the generalised MWC framework need a way of converting microscopic MWC constants into observed Adair-Klotz constants. Here, we derive equations that can be used to compute Adair-Klotz constants and apply them to two special cases of the generalised MWC model presented here.

### Obtaining Adair-Klotz constants from microscopic association constants for a protein with four non-equivalent binding sites

Consider a protein *P *with four binding sites for ligand *X*. The first apparent association constant, *K*_1 _is defined as follows:



where [*P*_0_] denotes the concentration of unbound protein, [*P*_1_] the concentration of protein with exactly one ligand molecule bound and [*X*] the concentration of ligand. Since *P *is an allosteric protein, it can exist in two different conformations: The high-affinity *R *conformation and the low-affinity *T *conformation. If we denote by [*R*_*i*_] the concentration of protein in the *R *state bound to *i *ligand molecules (and analogous for [*T*_*i*_]), we can re-write the above expression to



Since we treat the four binding sites as non-equivalent, we have to discriminate between them. The first ligand molecule bound to the protein in the *R *state can bind to either site *A*, *B*, *C*, or *D*. If *R*_*A *_denotes the concentration of protein in the *R *state bound to exactly one ligand molecule at site *A *(and analogous for sites *B*, *C*, and *D*, and for the *T *state), the above equation becomes:



The balance between unbound protein in the *T *and *R *states is given by the allosteric isomerisation constant, *L *(). We can now use this relationship and derive an equation that links the apparent first association constant *K*_1 _to the microscopic association constants ( for site *A *in the *R *state, and analogous for the other binding sites, and the *T *state):



Substituting for [*T*_0_] and simplifying, we obtain

(8)

In a similar manner we can consider the second association constant, *K*_2_



Again, distinguishing between the *R *and *T *states and between the four different binding sites, we obtain:



This reduces to:

(9)

We can apply the same reasoning to the third ligand binding event:



which eventually gives:

(10)

And, similarly for *K*_4_:

(11)

Note that in the case of four identical binding sites,  and , and the above expressions reduce to conversion equations for identical binding sites reported by Edelstein [7].

### Obtaining the *i*^*th *^Adair-Klotz constants from microscopic association constants for a protein with *n *non-equivalent binding sites

In general, for a protein with *n *ligand binding sites, we can express the apparent association constant for the *i*^*th *^binding event by computing the ratio between the concentrations of end products and initial reactants. The equation for the *i*^*th *^apparent association constant thus reads as follows:



As above, both [*P*_*i*-1_] and [*P*_*i*_] are sums of protein populations in two different states and with ligand molecules bound to combinations of different binding sites. We can again distinguish between *R *and *T *state, which yields:



If we now assume that the *n *ligand binding sites are, in general, non-equivalent, we must account for the fact that *R*_*i *_is a collection of protein molecules in the *R *state with all possible combinations of *i *out of *n *ligand binding sites occupied. In other words:

(12)

Expressing every *R*_*j*1*j*2...*ji *_in terms of [*R*_0_], [*X*] and the microscopic association constants, we can write *R*_*i *_in the following way:

(13)

Introducing the following abbreviations

(14)

(15)

we can obtain the expression for 



Now, again, we can use the relationship [*T*_0_] = *L *[*R*_0_] and eliminate [*X*]^*i *^and [*R*_0_] and obtain:

(16)

with  and  as defined above.

If the binding sites can be classed into *k *sub-groups that have the same affinity (*m*_1 _binding sites with affinity *m*_2 _binding sites with affinity , etc.), the expression for  can be written as follows:

(17)

In the next section, we will consider two proteins with four binding sites each, which constitute extreme cases: In the case of calmodulin, all binding sites are different, so the protein can be seen as having four sub-groups of binding sites containing one binding site each (*m*_1 _= *m*_2 _= *m*_3 _= *m*_4 _= 1). In the case of hemoglobin, all binding sites are equivalent, so there is only one sub-group of binding sites containing four elements.

### Allosteric model of calmodulin

To illustrate the practical relevance of these conversion equations we applied them to a previously proposed MWC model of calmodulin [8]. According to this model, calmodulin can exist in two different states, *R *(that corresponds to the open state, stabilised by binding of calcium) and *T *(that correspond to the closed, often mistakenly called "apo", state). Each of these states can bind four calcium ions. The four different binding sites were designated *A*, *B*, *C*, and *D *(*A *and *B *on the N-terminal domain, *C *and *D *on the C-terminal domain, with no sequential order being implied within the domains). Each of the states and each of the reactions was explicitly modelled, with distinct dissociation constants for each of the sites. The dissociation constants for the *R *state were  = 8.32 × 10^- ^^6 ^M,  = 1.66 × 10^- ^^8 ^M,  = 1.74 × 10^- ^^5 ^M, and  = 1.45 × 10^- ^^8^M. According to this model, *L *= 20670, and *c *= 0.00396 for all four binding sites [8]. The calmodulin concentration used for the model was 2 × 10^7 ^M [8], and simulations were run using COPASI [9].

When the fractional occupancy of calmodulin is plotted against initial free calcium concentration, simulation outcomes seem to agree quite well with experimental observations [8], but such a plot does not provide a direct way of quantifying this agreement.

To do this, we inserted the parameters of the MWC model into equations 8 to 11 to obtain Adair-Klotz constants. These can be compared to Adair-Klotz constants previously obtained in experimental studies [10-14], as listed in Table 1. This comparison shows that all four Adair-Klotz constants computed from the general MWC model lie within the experimentally reported range, and thus show that the MWC model is indeed consistent with experimental data.

**Table 1:** Apparent Adair-Klotz constants for the calmodulin model

	this paper	reported range
*K*_1_	5.1860 × 10^5^	1.16 × 10^5 ^[11] – 1.7 × 10^6 ^[11]
*K*_2_	5.1601 × 10^5^	1.4 × 10^5 ^[11] – 8.9 × 10^5 ^[12]
*K*_3_	1.3377 × 10^5^	2.86 × 10^4 ^[13] – 2.9 × 10^6 ^[11]
*K*_4_	3.8784 × 10^4^	1.7 × 10^3 ^[14] – 1.12 × 10^5 ^[13]

Apparent Adair-Klotz constants (in M) for the calmodulin model as computed with our method, and comparison to several experimental reports [10-14] and data reviews [11].

**Figure 1:**
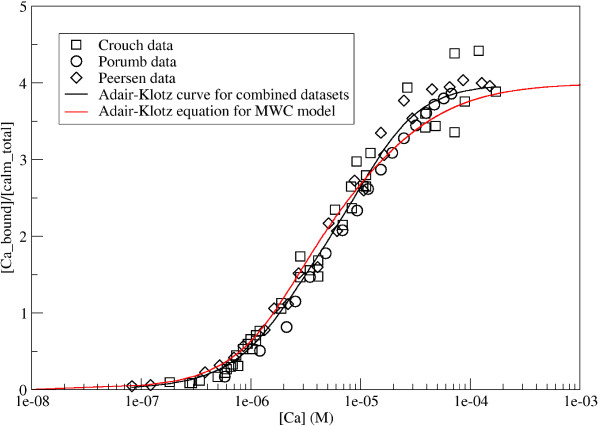
**Comparison of the calmodulin model with experimental data**. Red curve shows the Adair-Klotz equation using the Adair-Klotz constants obtained from the MWC model of calmodulin. Symbols are used to represent data points from various experimental measurements of calmodulin binding to calcium: Circles for Porumb [12], squares for Crouch and Klee [10], diamonds for Peersen *et al*. [15]. The black line represents a fit of all of these data set to the Adair-Klotz equation, which was obtained using the "Non-linear curve-fitting" function in grace .

Figure 1 visualises this comparison: The Adair-Klotz curve obtained from the MWC model is compared to experimental measurements done by Porumb [12], Crouch and Klee [10], and Peersen *et al*. [15] and to an Adair-Klotz fit to the combination of all three data sets. The plot illustrates that the Adair curve obtained from the parameters of the generalised MWC model presented here is similar to that obtained from experimental data, and that computing an Adair-Klotz function from the parameters of a MWC model does indeed provide a way of comparing an allosteric model to experimental measurements.

### Allosteric model of Hemoglobin

In a similar manner, the case in which all binding sites are equivalent [7] can be seen as a special case, in which there is only one sub-group of identical binding sites. With four binding sites, as is the case for hemoglobin, we obtain:

(18)

(19)

(20)

(21)

Yonetani *et al*. [16] fitted the same data for hemoglobin binding to oxygen once using the MWC framework and once using the Adair-Klotz framework. This study provides an excellent opportunity to test the validity of the conversion equations presented here: By using the results of their MWC fit and inserting *K*_*R*_, *K*_*T*_, and *L *into the equations presented in [7], we get an independent determination of the Adair-Klotz constants *K*_1 _to *K*_4_. Table 2 compares the Adair-Klotz constants thus obtained to the Adair-Klotz constants obtained by Yonetani *et al*. [16]. Both methods yield essentially the same results, slight differences are presumably due to rounding errors and/or to limitations of the data fitting algorithms used, as well as possible over-fitting in the case of the Adair-Klotz framework.

**Table 2:** Comparison of MWC and Adair-Klotz constants for hemoglobin

	this paper	Yonetani *et al*. [16]
	7.68 × 10^-3^	7.20 × 10^-3^
	0.96 × 10^-2^	1.05 × 10^-2^
	1.52 × 10^-2^	1.15 × 10^-2^
	2.32 × 10^-2^	2.33 × 10^-2^

Experimental and theoretical determination of Adair-Klotz constants (in torr^-1^) from MWC constants at pH 7.0. K_*R *_= 3.0 × 10^-2^torr^-1^, K_*T *_= 7.0 × 10^-3 ^torr^-1^, and *L *= 33, as obtained by Yonetani *et al*. by fitting data with an MWC equation [16]. We used these to compute *K*_1 _to *K*_4 _using the equations presented in [7] and here compare them to *K*_1 _to *K*_4 _obtained by Yonetani *et al*. by fitting the same data with an Adair-Klotz equation [16]. Note that Yonetani *et al*. used a slightly modified version of the Adair-Klotz equation, meaning that *K*_1 _in [16] corresponds to *K*_1 _in [4], *K*_2 _in [16] to *K*_2 _in [4], *K*_3 _in [16] to *K*_3 _in [4] and *K*_4 _in [16] to 4*K*_4 _in [4]. To allow easier comparability, we used Yonetani's notation for this table and labelled the constants , ...,  to avoid confusion with the original Klotz notation used everywhere else in this paper.

## Discussion and conclusion

The generalised MWC model proposed here opens up new ways of applying the allosteric framework: Not only to multimers consisting of identical subunits with one ligand binding site on each, but also to proteins with several binding sites of different affinities for the same ligand, be it multimers with more than one binding site on each subunit or monomeric proteins containing several binding sites. This framework has been used for an allosteric model of calmodulin [8], and could be useful in the analysis of a wide range of other proteins.

Other generalisations of the MWC framework have been presented in the past. Mello and Tu [17] have proposed a heterogeneous MWC (HMWC) model for allosteric proteins or protein complexes that bind to different types of ligand (but where there is only one affinity per ligand). This can easily be combined with the model presented here: The fractional occupancy for a generalised heterogeneous protein with two different types of ligand, and binding sites of different affinity for each ligand, would be:

(22)

where [*X*_1_] represents the first ligand, for which *n*_1 _binding sites exist, and [*X*_2_] the second ligand, for which there are *n*_2 _binding sites. For a heterogeneous complex with *m *types of ligands, the equation is

(23)

The case in which binding sites for a given ligand can be grouped into sets of same affinity is straight-forward, as is the computation of fractional occupancy, *R*.

Najdi *et al*. [18] have proposed a generalised MWC (GMWC) model for a protein binding to several ligand types and regulated by multiple allosteric activators or inhibitors. This model can be combined with the model presented here by replacing the term that denotes substrate concentration and affinity for each ligand in [18] by the appropriate sum: in the notation employed by [18], this would mean replacing  by  for each ligand. Such a combined model could then cater for proteins that bind to several ligand types (with non-identical binding sites per ligand) and that are regulated by multiple allosteric activators or inhibitors.

In biology, the same question can be tackled at different levels and with different approaches, often based on different underlying theoretical framework. These approaches, however, need to be comparable to allow for cross-validation and for the assembly of different types of data into a comprehensive understanding of a given process. For instance, computational modellers need a way of comparing their models with experimental results to assess the validity of their models. In particular, mechanistic models need to be comparable to data or to the phenomenological models describing them. We offer a way of relating intrinsic association constants in allosteric models to Adair-Klotz constants and thus to bridge the gap between generalised allosteric models and experimental observations.

Apart from enabling modellers to validate their models – as shown here in the two example cases – these conversion equations could also help in model construction by providing ways to constrain parameter space and facilitate the estimation of allosteric parameters, which is very useful in cases where there is little or no additional experimental evidence that could help with their derivation.

## Abbreviations

MWC: Monod-Wyman-Changeux; R: relaxed; T: tense.

## Authors' contributions

MIS designed the generalised MWC framework and wrote the conversion equations with the help of SJE. All authors contributed to the manuscript. All authors read and approved the final manuscript.

## References

[B1] Wyman J, Gill SJ (1990). Binding and linkage Functional chemistry of biological molecules.

[B2] Hill AV (1910). The possible effects of the aggregation of the molecules of hæmoglobin on its dissociation curves. J Physiol.

[B3] Adair GS (1925). The hemoglobin system. IV. The oxygen dissociation curve of hemoglobin. J Biol Chem.

[B4] Klotz IM (1946). The Application of the Law of Mass Action to Binding by Proteins. Interactions with Calcium. Arch Biochem.

[B5] Klotz IM (2004). Ligand-receptor complexes: origin and development of the concept. J Biol Chem.

[B6] Monod J, Wyman J, Changeux JP (1965). On the Nature of Allosteric Transitions: A Plausible Model. J Mol Biol.

[B7] Edelstein SJ (1975). Cooperative interactions of hemoglobin. Annu Rev Biochem.

[B8] Stefan MI, Edelstein SJ, Le Novère N (2008). An allosteric model of calmodulin explains differential activation of PP2B and CaMKII. Proc Natl Acad Sci USA.

[B9] Hoops S, Sahle S, Gauges R, Lee C, Pahle J, Simus N, Singhal M, Xu L, Mendes P, Kummer U (2006). COPASI-a COmplex PAthway SImulator. Bioinformatics.

[B10] Crouch TH, Klee CB (1980). Positive cooperative binding of calcium to bovine brain calmodulin. Biochemistry.

[B11] Burger D, Cox JA, Comte M, Stein EA (1984). Sequential Conformational Changes in Calmodulin upon Binding of Calcium. Biochemistry.

[B12] Porumb T (1994). Determination of calcium-binding constants by ow dialysis. Anal Biochem.

[B13] Shifman JM, Choi MH, Mihalas S, Mayo SL, Kennedy MB (2006). Ca2+/calmodulin-dependent protein kinase II (CaMKII) is activated by calmodulin with two bound calciums. Proc Natl Acad Sci USA.

[B14] Haiech J, Klee CB, Demaille JG (1981). Effects of cations on affinity of calmodulin for calcium: ordered binding of calcium ions allows the specific activation of calmodulin-stimulated enzymes. Biochemistry.

[B15] Peersen OB, Madsen TS, Falke JJ (1997). Intermolecular tuning of calmodulin by target peptides and proteins: differential effects on Ca2+ binding and implications for kinase activation. Protein Sci.

[B16] Yonetani T, Park SI, Tsuneshige A, Imai K, Kanaori K (2002). Global allostery model of hemoglobin. Modulation of O(2) affinity, cooperativity, and Bohr effect by heterotropic allosteric effectors. J Biol Chem.

[B17] Mello BA, Tu Y (2005). An allosteric model for heterogeneous receptor complexes: understanding bacterial chemotaxis responses to multiple stimuli. Proc Natl Acad Sci USA.

[B18] Najdi TS, Yang CR, Shapiro BE, Hatfield GW, Mjolsness ED (2006). Application of a generalized MWC model for the mathematical simulation of metabolic pathways regulated by allosteric enzymes. J Bioinform Comput Biol.

